# An Operation for Permanent and Quick Cure of Hemorrhoids, Almost Bloodless and Painless

**Published:** 1903-12

**Authors:** John C. McAfee

**Affiliations:** Macon, Ga.


### AN OPERATION FOR PERMANENT AND QUICK CURE OF HEMORRHOIDS, ALMOST BLOODLESS AND PAINLESS.*

BY JOHN C. McAFEE, A.B., M.D.,

MACON, GA.

I approach the subject of the surgical treatment of hemorrhoids fully aware that it has been discussed and rediscussed and talked about generally, from time immemorial, as much as, and probably more than, almost any subject in the field of surgery, and the subsequent remarks in this paper, with the results described, can be my only apology for bringing up the subject at this juncture. The title of my paper is intended to cover only the internal variety of this trouble.

Dr. J. P. Tuttle, of New York, whom we had the pleasure of

* Read before the Georgia Medical Association, April, 1903.

hearing before this Association in 1899, and whose authority as a rectal specialist is generally recognized, makes the sweeping statement that "seventy-five per cent. of the human race who reach the age of fifty years suffer at some time to a greater or less degree from hemorrhoids."

We all know the universality of this trouble, and it follows as a natural sequence that the profession is called upon in no uncertain tones for its relief. I shall not discuss the etiology of hemorrhoids, as none has yet been established as being a certain cause in all cases. In one instance alcohol seems to play a part, but on the contrary we have all known the most pronounced sots who were absolutely free from the disease. Indoor and sedentary habits are sometimes put down as causes, and likewise long hours of standing, heavy lifting, etc., yet we know people by the score following all these occupations who escape absolutely. No cause with which I am acquainted can be set down as a producer of hemorrhoidial disease in every case in which such etiologic factor is present. Fortunately for us, it is not necessary to discover the cause of at least ninety-nine per cent. of all cases in order to cure them absolutely.

I shall take the liberty of quoting from some very distinguished writers and workers in this field of surgery, to substantiate the statement that all methods of operating for internal hemorrhoids so far announced are imperfect and unsatisfactory, and that the active workers are striving for improvement in this line.

The following is from Tuttle, of New York: "The great interest surgeons take in the treatment of hemorrhoids is due largely to the fact that all methods so far devised are more or less imperfect. The ligature method is tedious, painful, slow in healing, and if statistics can be relied upon, is the most dangerous to life of all the accepted surgical procedures in this disease. The clamp and cautery, while efficient and satisfactory in the end, is not an ideal surgical method, for it leaves an open granulating wound which requires three weeks or longer to heal; and in inexpert hands this, like the ligature, may be followed by protracted ulceration and strictures. Finally," says the same author, "the Whitehead operation is too difficult a procedure for the occasional operator to undertake, and when improperly performed is liable to give the worst final results of any."

These are the words of no tyro in surgery, nor occasional operator, but they come direct from one of the most distinguished rectal specialists of the present day, and one known to you all.

Dr. Tuttle pointedly condemns the ligature method as being "the most dangerous to life of all the accepted surgical procedures in this disease," and yet Allingham, of England, says of the clamp and cautery: "As regards danger to life, as far as my most careful researches have led me to a conclusion, it is quite six times as fatal as the ligature." Matthews, of Louisville, in discussing the clamp and cautery method, says: "It cannot be denied that the burning of this amount of tissue causes a very great deal of pain after the operation. No one can say that the iron has had full cautery effect upon every vessel, and therefore hemorrhage is more likely to occur than after the ligature. The period of convalescence is very long; frequently more sloughing of the tissues than was intended takes place; and we all know how natural it is for extensive scar tissue to follow burns; therefore contraction of the anus and rectum is to be feared." The same author says: "I cannot see the advisability of using the clamp and cautery for the removal of hemorrhoids except in a few selected cases." What meaneth all this outcry against these time-honored procedures in the treatment of this most annoying, and often dangerous, ailment, which claims three out of every four, on an average, of human beings for its victims? And the complaint is made, too, not by inexperienced operators, not even by general surgeons only, but by the masters in this special field. Therefore, no one of us has a vestige of right to bob up and say the fault was with the operator, and not the operation. These methods have been faithfully tried, by competent men, and found unsatisfactory, in many instances dangerous and very troublesome, and some of the most skilled operators are the loudest in their condemnation of one and another of the methods.

The plan of injecting hemorrhoids yields beautiful results in some selected cases, and yet these same leading surgeons warn us to beware of its use, as it is fraught with danger and occasionally proves fatal, but more frequently results in abscess with sloughing. It will be advisable only in selected cases.

Now, having thus reasonably and for good reasons, practically discarded all these methods of treating hemorrhoids, and having on our hands so large a percentage of the human race as sufferers from the trouble, what shall we do? I have a method to propose that is not original, except in its application and some of its details. I saw Dr. S. T. Earle, of Baltimore, do his operation in the winter of 1897-8. His is a modified Whitehead operation, which is not at all the operation of excision which I shall presently describe to you. Dr. C. S. Parkhill, of Hornellsville, New York, claims priority in the excision-with-immediate-suture operation. Dr. Parkhill states that his first operation of this kind was done on June 11, 1898. I saw Earle do the same operation in the winter of 1897-8, and hence he is entitled to priority in this operation. The operation I shall now describe to you briefly is, as I have said, not original with me, except in its application. The patient is etherized, the sphincter thoroughly divulsed, and the hemorrhoids are removed one by one, using the clamp devised by Dr. Earle of Baltimore. The clamp, which I show you, is applied to the base of the pile in its linear axis at junction of normal mucous membrane, and locked. Then, using a double-thread catgut suture, Lee's No. 2 catgut, I begin sewing under the clamp at the muco-cutaneous junction, catching the two distal ends of my thread in a forceps. After the needle is drawn through under the pile clamp, one of the threads on the needle side is cut off to facilitate tying. Then, cutting off the pile above the clamp, a continuous suture is carried round and round the clamp, proceeding toward the upper end of the pile tumor. When the upper end is reached, it is tied, the clamp withdrawn, the suture tightened, and the lower end tied. This procedure is then carried out with each pile tumor till all are removed. I have performed this operation in about twenty-five cases, and never yet have I seen the semblance of a complication, and my results have been perfect, so far as I know, and I have kept track of nearly all my cases. The only objection I can see so far to the operation is that it requires some delicacy of manipulation in its performance. After the operation is completed, only line of suture remains where the pile tumor was previously situated, and the approximation of the cut edges is perfect. The loss of blood is practically nil, and I have never seen the slightest degree of disturbance from after contraction of the anus, and generally about four tumors are removed in each case. An anodyne is often not needed after the operation, and the patients invariably tell me they feel better on the day following than on the day preceding the operation. A patient can return home in one week after the operation, and they usually resume their regular avocations by the tenth day. What more can be said in favor of an operation for hemorrhoids?

Many of the operators of our own city have seen this method applied, and favor it very much, as all its points are preferable to the clamp and cautery or ligature. There is no sloughing, as in the clamp and cautery method, and no constriction of tissues, as in the ligature method, but instead, a neatly approximated and sutured incision, which, in my experience, heals kindly and promptly, and the cure is radical.

